# Monitoring peroxisome dynamics using enhanced green fluorescent protein labeling in *Alternaria alternata*

**DOI:** 10.3389/fmicb.2022.1017352

**Published:** 2022-10-25

**Authors:** Ziqi Lu, Jian Guo, Qiang Li, Yatao Han, Zhen Zhang, Zhongna Hao, Yanli Wang, Guochang Sun, Jiaoyu Wang, Ling Li

**Affiliations:** ^1^The Key Laboratory for Quality Improvement of Agricultural Products of Zhejiang Province, College of Advanced Agricultural Sciences, Zhejiang Agriculture and Forestry University, Hangzhou, China; ^2^State Key Laboratory for Managing Biotic and Chemical Threats to the Quality and Safety of Agro-Products, Institute of Plant Protection and Microbiology, Zhejiang Academy of Agricultural Sciences, Hangzhou, China; ^3^College of Food and Health (College of Modern Food Industry), Zhejiang Agriculture and Forestry University, Hangzhou, China

**Keywords:** *Alternaria alternata*, eGFP-PTS2, peroxisome, dynamic analysis, lipid

## Abstract

Brown leaf spot on tobacco is a serious fungal disease caused by *Alternaria alternata*. Peroxisomes are organelles playing an important role in the development and infection of plant pathogenic fungi. But, until now, there is no report on the peroxisome dynamics during the conidia germination of *A. alternata*. To evaluate the roles of peroxisome in the development of the fungus, in the present work, an enhanced green fluorescent protein (eGFP) cassette tagged with peroxisome targeting signal 2 (PTS2) was integrated into *A. alternata* to label the organelles, and an eGFP cassette carrying a nuclear located signal (NLS) was performed parallelly. The transformants containing the fusions emitted fluorescence in punctate patterns. The fluorescence of eGFP-PTS2 was distributed exactly in the peroxisomes while those of eGFP-NLS were located in the nucleus. Typical *Aa*GB transformants were selected to be investigated for the peroxisome dynamics. The results showed that during spore germination, the number of peroxisomes in the spores decreased gradually, but increased in the germ tubes. In addition, when the transformants were cultured on lipid media, the numbers of peroxisomes increased significantly, and in a larger portion, present in striped shapes. These findings give some clues for understanding the peroxisomal functions in the development of *A. alternata*.

## Introduction

Tobacco (*Nicotiana tabacum* L.) is an annual or perennial herbaceous plant of *Solanaceae* and an important cash crop with leaves of high commercial value. Both the planting area and the output of tobacco in China rank first in the world (Huang et al., 2021). Brown spot is one of the most severe diseases of tobacco caused by *Alternaria alternata* (Fr.) Keissler (Main, 1969; Dobhal and Monga, 1991), during the growing season, especially the mature stage (Zhang et al., 2011). The typical symptom of the disease on tobacco leaves is brown necrotic spots surrounded by yellowish-green halos. The disease normally causes broken leaves, and even affects the color and thickness of the baked leaves, resulting in the huge economic loss (Jenning et al., 2002; Slavov et al., 2004; Yakimova et al., 2009; Chen et al., 2017). During the process of infection, *A. alternata* forms a special infection structure called appressorium, a specialized differentiated structure that can firmly attach to the leaves (Hatzipapas et al., 2002; Wang, 2019).

The reactive oxygen species (ROSs) formed in host cells is the main barrier to fungal invasion and establishment of parasitism (Barna et al., 2003). The pathogens have to suppress the ROSs for successful infection. The enzymes required in ROSs degradation are distributed in peroxisomes in large portions. It was proved that pathogens generated an increased number of peroxisomes in response to the oxidative stress generated by the host (Chen et al., 2017). The metabolic reactions in the peroxisomes and the genes involved in peroxisomal biogenesis were demonstrated related to the pathogenicity of plant fungal pathogens. Peroxisomes plays important roles in hyphal growth, conidiation, conidial germination, and development of infection structures (Ramos-Pamplona and Naqvi, 2006; Kubo et al., 2015; Li et al., 2017; Chen et al., 2018; Wang et al., 2019; Falter and Reumann, 2022). The peroxisomal dynamic in *A. alternata* during infection is helpful for a better understanding of the pathogenesis of the fungus. However, there is a little knowledge on this topic (Pliego et al., 2009; Wang et al., 2015). Fluorescent proteins are widely used to assess gene expression and monitor the proteins’ cellular or subcellular distribution (Yang et al., 2018). To date, three fluorescent proteins [enhanced green fluorescent protein (eGFP), red fluorescent protein (RFP), and yellow fluorescent protein (YFP)] have been mainly used in fungi (Straube et al., 2005).

In the present work, we used a fusion of eGFP with PTS2, driven under the promoter of *MPG1* gene (MGG_10315) from *Magnaporthe oryzae*, to monitor the peroxisomes in *A. alternata*. Using the *Agrobacterium tumefaciens*-mediated transformation (*At*MT), the eGFP-PTS2 was integrated and expressed stably in the transformants. The dynamic changes of peroxisomes in the spore germination of *A. alternata* were observed by fluorescence confocal microscopy. Meanwhile, we marked the nucleus of *A. alternata* using an eGFP version tagged with a nuclear located signal (NLS) and compared the relative locations of the nucleus and peroxisomes in the spores. These results provide a framework for the study of the pathogenic mechanisms and organelles biology of *A. alternata*.

## Materials and methods

### Fungal species and culture medium

We isolated *A. alternata* wild-type strain C15 from leaves with typical symptoms of tobacco brown spots and stored the strain in our laboratory. The wild-type strain and all transformants were cultured on a complete medium (CM) at 28°C for 3–10 days. The strain of *A. tumefaciens* used was *AGL1*. YEB agar and liquid medium were prepared as described by Holsters et al. (1978). YEB containing 50 μg/ml kanamycin, 34 μg/ml rifampicin, and 50 μg/ml ampicillin was used to culture *A. tumefaciens*. Induction medium (IM) (solid) was used to co-culture *A. alternata* and *A. tumefaciens* (Michielse et al., 2008). CM plates containing 50 μg/mL hygromycin B (Roche, Mannheim, Germany) was used to screen the transformants. The wild-type and transformants strains were inoculated on a minimal medium (MM) supplemented with 0.5% (v/v) Tween 80, 1% (v/v) olive oil, or 1% (v/v) glycerin, and cultured at 28°C for 7 days to compare the ability of the strains to utilize the carbon sources (Li et al., 2017).

### Construction of fluorescent fusion vectors

p1300HMGB containing the *HPH* gene and eGFP-PTS2 (a PTS2-tagged eGFP), abbreviated as pHMGB in the present work, were used as peroxisome markers. To construct the pHMGB vector, we used p1300BMGB, a vector carrying the glufosinate-ammonium resistance gene (*BAR*), and eGFP-PTS2 with the promoter of *MPG1* gene (MGG_10315) from *M. oryzae* (abbreviated as pBMGB in present work) (Wang et al., 2008; Li et al., 2014). A 1.36 kb fragment of the *HPH* cassette was amplified using p1300-KO as the template and the primer pair *HPH*-Xh1/*HPH*-Xh2. We replaced the *BAR* gene in pBMGB with the *HPH* cassette using *Xho*I digestion to generate pHMGB. All the primers used are listed in Table 1. pHMGB was used to monitor the peroxisomes in *A. alternata*. pRp27GFP-NLS was generated by fusing a NLS fragment to the C terminus of eGFP, which was driven by a constitutive promoter from *M. oryzae* ribosomal protein 27 gene (RP27) in the binary vector pCAMBIA1300. All the vectors were transformed into *A. alternata*, respectively, using the *At*MT method (Rho et al., 2001).

**TABLE 1:** Hygromycin B amplification primer.

Primer	Sequence	Length
HPHCK1	TTCGCCCTTCCTCCCTTTATTTCA	1.0 kb
HPHCK2	GCTTCTGCGGGCGATTTGTGTACG	

### *Agrobacterium tumefaciens*-mediated transformation of *Alternaria alternata*

*AGL1* carrying pHMGB or pRp27GFP-NLS was spread on an LB agar plate containing kanamycin (50 mg/L) and incubated for 2 days at 28°C. A single colony was cultured in 5 mL of LB broth containing 50 mg/L kanamycin at 28°C with a shaking speed of 200 rpm for 48 h. Cells of 2 mL culture were then collected by centrifugation (5,000 × *g*) for 10 min, washed using *A. tumefaciens* induction medium (AIM, lipid) for 2 min, and then diluted, respectively, into OD_600_ about 0.6 in AIM supplemented in 200 mM acetosyringone (AS) and an AS-free AIM as a control. For transformation, 100 μL of *A. tumefaciens* cells were mixed with 100 μL 1 × 10^6^ of *A. alternata* conidia, the mixture was spread on a sterilized nitrocellulose filter membrane (Pore size 0.45, Φ50 mm, Whatman, Sangon, Shanghai, China) overlaid on the surface of AIM agar plates and incubated for another 48 h at 23°C in the dark. After the incubation, the nitrocellulose filter membrane was cut with a sterilized knife into strips and transferred into selective CM plates containing 200 μg/mL hygromycin B, 200 μg/mL cefotaxime sodium, 200 μg/mL tetracycline hydrochloride and incubated for 5–7 days to selected the transformants. Thirteen selected hygromycin B-resistant transformants were verified by growing in a new selective CM plate for another 3–4 days at 28°C, together with the wild-type as a control.

### Analysis of *Alternaria alternata* transformants

We used polymerase chain reaction (PCR) to amplify the *HPH* gene (with primers HPHCK1 and HPHCK2; Table 1). The amplified fragment of *HPH* was 1.0 kb long. We used a 50 μL reaction volume containing 2 μL Taq, 4 μL dNTP, 5 μL 10 × buffer, 35 μL ddH_2_O, 2 μL forward primers, and 2 μL reverse primer. Our PCR reaction conditions were 5 min at 95°C; 35 cycles of 30 s at 95°C, 30 s at 55°C, and 90 s at 72°C; and 10 min at 72°C. PCR products were held at 4°C (Li et al., 2014).

### Measurement of colony growth and spore production

The mycelia of the wild-type *A. alternata* strains and the 13 transformations were picked out and inoculated on a 9 cm CM culture medium, respectively, which was cultured at 28°C for 10 days under darkness. The experiment was repeated three times for each colony. Then, each plate was washed with 5 mL of sterile water, and the collected solution was filtered through three layers of lens paper. Collected spores were counted using a hemocytometer, and the conidia concentration and production were calculated.

### Confocal microscopy and calcofluor white staining

We used a Leica SP2 confocal microscope (Leica, Germany) and a ZEISS LSM780 inverted confocal microscope (Zeiss, Germany) to examine transformant hyphae and spores. We used an excitation wavelength of 488 nm, and an emission wavelength of 520 nm. And we also observed the peroxisome situation of four mediums (MM, MM + 0.5% (v/v) Tween 80, MM + 1% (v/v) olive oil, or MM + 1% (v/v) glycerin) under confocal microscopy. Each treatment sets three replications with 20 conidia per replicate (*n* = 60).

The concentration of conidia solution of *A. alternata* was adjusted to 1 × 10^5^ number/mL, and 20 μL of spore droplets were placed on both plastic coverslips and the onion epidermis and cultured under 28°C dark moisturizing conditions. Then, the spore solutions of 2, 4, 6, 8, 10, 12, 24, and 48 h were taken, respectively, and the dynamic changes of peroxisomes in the progress of spore germination and appressoria formation of *A. alternata* were observed by the fluorescence confocal microscopy. Each post-incubation hour set three replications. In addition, with 10 conidia, germ tube or appressoria per replicate were observed on plastic coverslips (*n* = 30).

Calcofluor white (CFW) staining using Fluorescent Brightener 28 (10 mg/ml, Sigma-Aldrich, Saint Louis, MO, USA) for the microscopy of mycelial branches was performed as described by Harris et al. (1994).

## Results

### Acquisition of pHMGB transformant in *Alternaria alternata*

**FIGURE 1:**
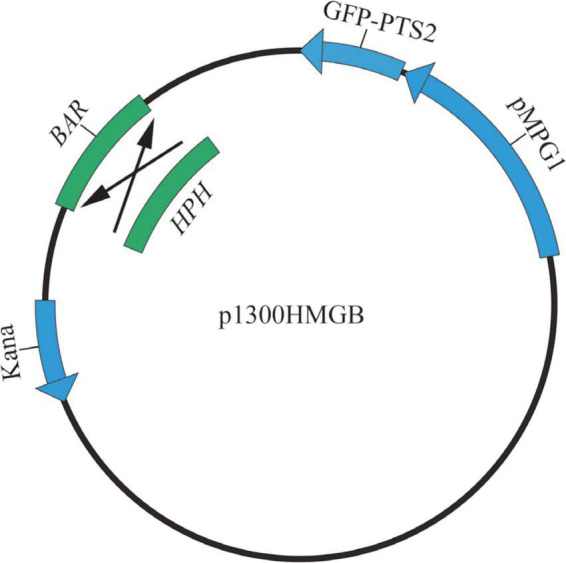
The structure of p1300HMGB.

The structures of pBMGB and pHMGB are shown in Figure 1. By the *At*MT method, we obtained 25 transformants for pHMGB. These transformants grew normally on the CM plates compared with the wild-type strain. After five successive subcultures without selection pressure, all 25 transformants were able to grow on selective CM but not the wild-type, indicating that all the obtained transformants are stable under our experimental conditions.

**FIGURE 2:**
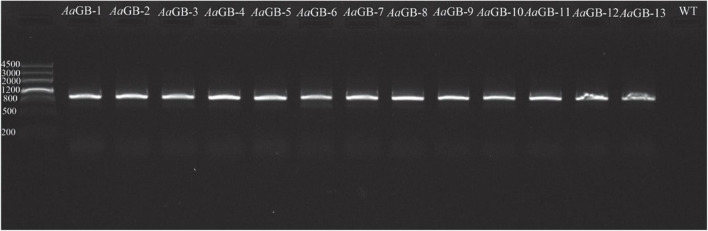
The result of transformants amplification hygromycin B by PCR.

Then, 13 transformants were randomly selected and confirmed by PCR amplification with the primers HPHCK1 (forward) and HPHCK2 (reverse). The hygromycin B gene can be amplified in transformants strains, but not in the wild-type strain C15. These results indicate that the pHMGB vector had been successfully inserted into the genome of the transformants (Figure 2).

### Enhanced green fluorescent protein labeling of peroxisome and nucleus in *Alternaria alternata*

**FIGURE 3:**
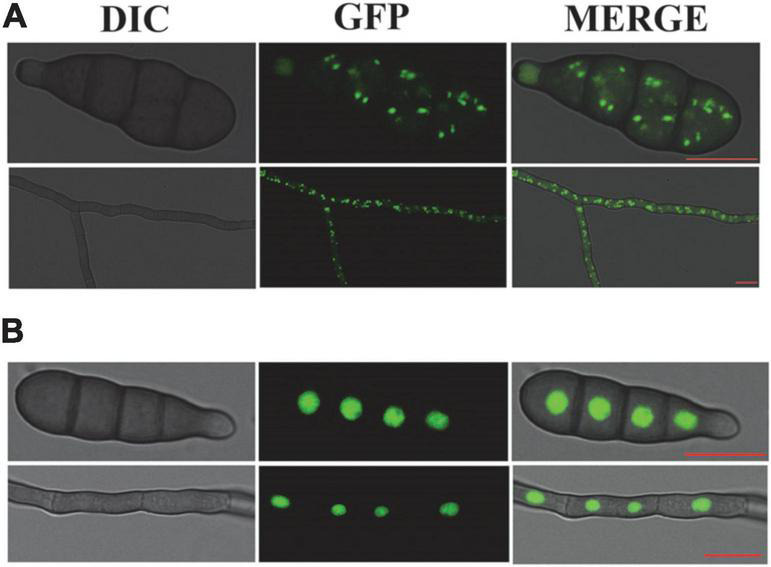
AaGB fluorescent labeling of the peroxisome with eGFP **(A)** and NLS fluorescent labeling of the nucleus with eGFP **(B)**, bar = 10 μm.

After the establishment of auxotrophic makers *via* the *At*MT, we were able to introduce a plasmid containing the maker gene hygromycin B and eGFP under the strong constitutive promoter *MPG1* or *Rp27*. Inspection of non-transformed *A. alternata* hyphae revealed strong auto-fluorescent in some compartments under a fluorescence microscope. In order to distinguish specific eGFP signals from such auto-fluorescent, eGFP was C-terminally tagged with PTS2 or a NLS from *A. alternata*. To quantify the transformation efficiency, we screened transformants microscopically. After 24 h incubation at 28°C in CM medium, 13 out of 25 eGFP-PTS2 transformants and 9 out of 14 eGFP-NLS transformants showed bright green fluorescence under a laser-scanning confocal microscope. However, no fluorescence was observed in the *A. alternata* wild-type strain C15 (control). We found the dots fluorescence are presented in hypha and spores of transformants. In *Aa*GB, green fluorescence is distributed in small green dots (0.2–1 μm in diameter) throughout the cell. We select a typical transformant *Aa*GB-1 to observe the dynamic of peroxisome in the progress of germination. The location and size of the small green dots are consistent with that of the peroxisome (Figure 3A). In eGFP-NLS transformants, they were visualized as spherical structures in the center of mycelia and conidial cell, where the cell nucleus is located (Figure 3B). These results indicate that the GFP fused with PTS2 or NLS is located in peroxisome or cell nucleus, respectively.

### Peroxisomal dynamics of *Alternaria alternata* during spore germination and appressoria formation

**FIGURE 4:**
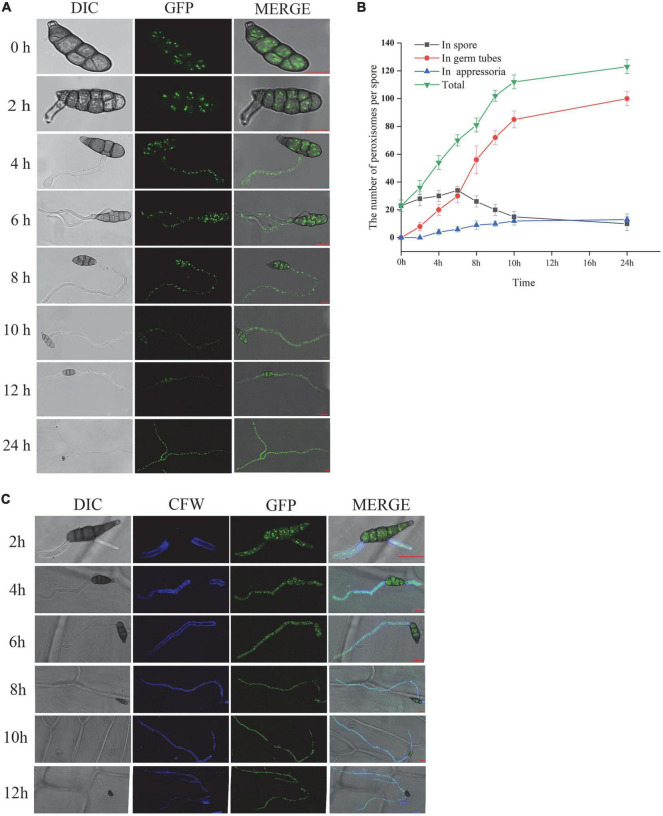
AaGB dynamic change of peroxisome by fluorescence confocal microscopy **(A)**, the number change of peroxisome **(B)**, and dynamic changes of the spore germination process by calcofluor **(C)**, bar = 10 μm.

To explore the role of peroxisome in spore germination and the infection process of *A. alternata*, we observed the dynamic changes in peroxisome by fluorescence confocal microscopy, placed on both plastic membrane and the onion epidermis at 0, 2, 4, 6, 8, 10, 12, and 24 h, respectively. The fluorescence in the eGFP-PTS2 transformants cells is punctate and mostly located throughout the cell at 0 h. During the elongation of the germ tube, the fluorescence intensity in the germ tube increased gradually. The number of peroxisomes in conidia, germ tubes, and appressoria was monitored within 24 h. The number of peroxisomes in the spore kept increasing before 6 hpi and decreased after 6 hpi, and increased continuously both in germ tube and appressoria (Figures 4A,B).

Calcofluor white can be combined with the cellulose and chitin of the fungal cell wall, so we can see the blue fluorescence under the fluorescence microscope. Using calcofluor white to stain the conidia solution of AaGB strain, which transformed the pHMGB into *A. alternata* at 2, 4, 6, 8, 10, and 12 h, we observe the structural dynamic changes in the conidia germination process (Figure 4C).

### Peroxisomal response to lipid stress

**FIGURE 5:**
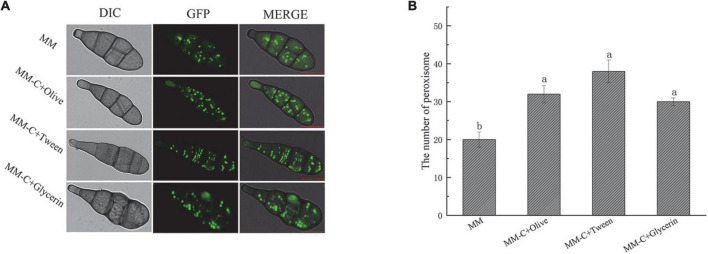
AaGB Lipid induced peroxisome fluorescence images **(A)** and changes of number **(B).** These images were filmed by laser confocal fluorescence microscope. AaGB is cultured for 7 days at 28°C, bar = 10 μm.

To observe the effect of lipid on peroxisome, *Aa*GB is inoculated on MM, MM-C + olive, MM-C + glycerin, and MM-C + Tween culture medium, which are cultured for 7 days at 28°C and then observed by laser confocal fluorescence microscope (Figure 5A). The number of peroxisomes is increased in the conidia upon the strains cultured on the other three media compared to those on the MM media. On MM media, the number of peroxisomes is 20, and on the other three media, the numbers of peroxisome are 32, 30, and 38, respectively (Figure 5B). This result indicates that the addition of the appropriate amount of lipids to the medium promotes the division of peroxisomes, thus increasing the number of peroxisomes. In addition, the peroxisome exhibited a strip morphology on Tween media, while exhibiting a punctate pattern on other lipid media (Figure 5A).

## Discussion

Previous studies have shown that peroxisomes are involved in fungal growth, development, sporulation, invasion, and parasitizing of several pathogenic fungi (Kubo et al., 2015; Li et al., 2017; Wang et al., 2019; Falter and Reumann, 2022). Visualization of peroxisomes did not affect their functions or dynamics. It only required expression of the fusion gene encoding PTS1 or PTS2 added to the C- or N-terminus of GFP, respectively (Goto-Yamada et al., 2022). This also accords with our observations, which showed that there was no significant difference in growth morphology, appressorium formation, and spore production between transformants and WT strains (Supplementary material). Direct observation of peroxisomal dynamic in *A. alternata* facilitates the understanding of the pathogenesis of brown spot disease. Because it provides useful information about peroxisome dynamics, such as their morphology, number, size, intracellular distribution, direction of movement, and interactions with other subcellular components, which was difficult to obtain by traditional approaches. Moreover, the fusion gene of GFP with NLS can also be used to screen the genes related to plant disease resistance, elucidating the mechanism underlying the interaction between plants and pathogens (Geng et al., 2021).

The results of this study showed that the transformants successfully expressed the peroxisome or nucleus marker eGFP-PTS2 or NLS, respectively. To illustrate the dynamic of peroxisome during spore germination, the labeled peroxisomes were observed using a laser-scanning confocal microscope. As shown in Figure 4B, the number of peroxisomes in the spore first increased and then decreased after 6 h incubation, while the number of peroxisomes in the germ tube kept increasing. A possible explanation for this might be that there is a high rate of fatty acid degradation by peroxisomal β-oxidation to produce the energy needed for germination, reducing the amount of lipid droplets, and the number of peroxisomes located in close proximity to lipid droplets eventually decreased as well. In addition, peroxisomes tended to gather at spore septa. Similar results were obtained in other species such as *M. oryzae* (Wang et al., 2008) and *Fusarium graminearum* (Seong et al., 2008). Wang et al. (2008) found that the number of peroxisomes increased rapidly during the first 2-h of germination, and researched the maximum number at 2 h post-incubation (hpi). Then, the peroxisomes in spores transferred to the infant appressoria, and the number of peroxisomes in *M. oryzae* spore decreased (Wang et al., 2008). Interestingly, as described by Seong et al. (2008), peroxisomes also tended to be concentrated at spore septa, and the abundance of peroxisomes in the asexual spores of *F. graminearum* is correlated with a high number of lipid droplets in their vicinity, which disappear during germination. Peroxisomes are essential for the formation of fruiting bodies and the maturation and germination of spores. They facilitate the utilization of reserve compounds *via* fatty acid β-oxidation and the glyoxylate cycle, producing the energy, acetyl-CoA, and carbohydrates needed for the synthesis of cell wall polymers and turgor generation in infection structures (Peraza-Reyes and Berteaux-Lecellier, 2013; Falter and Reumann, 2022). In the rice blast fungus, lipid droplets are translocated through septa from the conidial cells to the appressorium. It is possible to hypothesize that peroxisomes are localized near the spore septa to facilitate fatty acid breakdown and energy mobilization at the site of need. As shown in Figure 3, the location and size of small green dots are consistent with that of the peroxisome, exhibiting an elliptic peroxisome morphology. In eGFP-NLS transformants, eGFP visualized round nuclei, consistent with previous studies (Escano et al., 2009; Shoji et al., 2010). RP27 from *M. oryzae* was used as a promoter to construct the NLS vector pck128 and localized in nuclei during interphase (Jones et al., 2016). To our knowledge, it is the first time that the promoter RP27, which is from *M. oryzae*, also worked in the vector pRp27GFP-NLS when transformed into *A. alternata*.

It is well-known that peroxisomes plays an important role in lipid metabolism. The results of the current study showed that the number of peroxisomes markedly increased in the strains grown on a fatty acid medium compared with those grown on a minimum medium. The finding reflects that of Imazaki et al. (2010) who also found that the number of peroxisomes increased in hyphal cells grown on oleic acid compared with those grown in a glucose medium. In addition, there was an interesting finding that the peroxisome exhibited strip morphology on Tween media compared with a punctate pattern on other lipid media. Peroxisomes multiply by growth and division from preexisting organelles. Peroxisome fission is preceded by the elongation of the peroxisome membrane *via* Pex11 family proteins (Navarro-Espíndola et al., 2020). The possible explanation for this discrepancy might be that peroxisome is in the process of being divided on Tween media, and division has been completed on other lipid media.

In conclusion, the results from our study confirmed that eGFP combined with PTS2 and NLS can be integrated into *A. alternata* to label organelles. Expressing N-terminal GFP-tagged proteins can be used to verify the organelle localization of some secondary metabolites’ biosynthesis-related genes (Imazaki et al., 2010). Therefore, the labeling of fungal strains with fluorescent proteins is an effective way to elucidate the mechanisms underlying peroxisomal physiological activity in fungi and plants (Motley and Hettema, 2007; Goto-Yamada et al., 2022). The findings of this study have some important implications for future research, such as the co-localization of the nucleus and peroxisome using multi-color fluorescent proteins and the analysis of the gene involved in peroxisome assembly.

### The colony morphology and conidiation of transformants

To observe the colony morphology difference of transformants compared with wild-type strain, mycelial blocks are incubated in CM plates for 10 days at 28°C under light conditions. The colonies were observed and photographed each day. As the number of days of incubation increased, the colonies became progressively larger and by the 10th day the colonies had grown to cover the entire plate. There were no significant differences in the colony morphology between the transformants and the wild-type strain (Supplementary Figure 1A). Colonies are irregularly rounded adaxially dark brown, loose and obvious ring tread. The edges are obvious, and peripheral yellow halo is narrow or not obvious. A dark brown or black mold can be seen at the center of the colony (Supplementary Figure 1A). Sporulation is an important condition to measure the viability of a strain. The conidia production of all the transformants are similar compared with the wild-type strain. Conidiation of wild-type are 1.85 × 10^8^ cells/plate, and the transformants are from 1.73 × 10^8^ to 2.16 × 10^8^ cells/plate. Thus, it can be explained that the amount of sporulation of *At*MT gene transformed by *A. alternata* will not be affected basically (Supplementary Figure 1B).

### The colony growth speed of transformants

To measure the growth speed and development of transformants strain, we choose one of the strains (*Aa*GB-1) to measure the colony diameters for 10 consecutive days. On the first day of culture, *Aa*GB-1 grew at a faster rate. On the 5th of culture, the growth diameter was nearly 6 cm (Figures 4A,B). In addition, the *Aa*GB-1 strains cover the whole 9 cm plate on the 9th day (Supplementary Figures 2A,B).

### The conidia development progress of transformants

To observe conidia formation progress of transformations, we culture the *Aa*GB strains under 12 h light/12 h dark at 28°C for 5 consecutive days. The color of conidia is dark brown, which is formed by conidiophore and is borne singly or in bunches. With the increase in incubation time, the conidiophores will gradually elongate, branch, and form mycelium.

To observe the growth dynamic of conidia, the transformants strain are incubated in CM medium which is obliquely inserted into the cover glass at 25°C in the dark. The cultures are observed every 12 h from 0.5 to 5 days. During the first 2.5 days, the conidiophores continuously extend forward and branch. At the same time, only one conidium is formed at the apex of the branch, whose size and DNA contents increased. The conidia are inverted rod-shaped, with a transverse septum and longitudinal diaphragm on the surface, where the transverse septum is thicker and the number of transverse septa is mostly three. With the increase of culture time, the melanin production of conidiophores and conidia increased. New conidia constantly sprout from clusters of the old conidia, whose color is light in the initial period and melanin gradually deepens. Accordingly, the conidiophores branch continuously to give rise to clusters of conidia. At this point, a vast amount of conidia are produced, which are the main forms of infecting the host (Supplementary Figure 3).

### The conidia germination and appressorial formation of transformants

The conidia of the transformants *Aa*GB were incubated on a plastic membrane to allow appressoria formation for 24 h. The spores gradually germinate and grow sprouting tubes at 28°C in dark conditions. The conidia can grow unilaterally out of the shoot tube and from multiple sides. Under light microscopy, the lengths of the bud tube are similar to these conidia and the rate of the conidia germination is about 30% at the first 2 h, there were already transverse compartments and longitudinal diaphragms within the conidia. The attachment cell is the spores of the pathogenic fungus that germinate and expand at the tip of the budding tube, forming a special morphological structure similar to a sucker. Appressoria are first formed at 4 h, and the rate is only about 3%. As the incubation time increases, the bud tube of spore germination gradually elongates as well as the rate of conidia germination and appressoria formation increased continuously. The rate of conidia germination is about 95% and appressoria formation is nearly 50% at 12 h. In addition, the rate of conidia germination and appressoria formation is raised slightly at 24 h compared to 12 h. At 24 h, the rate of germination and appressoria formation is at their highest, and the elongated budding tubes began to branch and continue to elongate (Supplementary Figures 4A–C).

## Data availability statement

The original contributions presented in this study are included in the article/Supplementary material, further inquiries can be directed to the corresponding authors.

## Author contributions

JW: conceptualization. LL, ZZ, and JG: data curation. ZL and QL: formal analysis. LL, JW, and GS: funding acquisition. ZL and YH: investigation. ZZ and JW: methodology. YW and GS: project administration. JG and ZZ: software. JW and GS: supervision. ZL, LL, and ZH: validation. LL and JG: writing—original draft. JW and LL: writing—review and editing. All authors contributed to the article and approved the submitted version.

## Supplementary material

Supplementary Figure 1 PHMGB-Aa transformant colonies and wild-type morphology **(A)** and their sporulation **(B)**. These colonies and wild-type were obtained by culturing in CM medium under dark conditions of 25°C for 7 days.

Supplementary Figure 2 AaGB colony growth morphology changes **(A)** and growth diameter of the colony **(B)**. Observed and recorded continuously for 10 days at 28°C under light conditions.

Supplementary Figure 3 AaGB transformant spore growth dynamic. The growth dynamics of spores in 5 days under 25°C light conditions were observed and recorded.

Supplementary Figure 4 Observation of the dynamics of AaGB conidial germination **(A)** and its germination rate **(B)**, appressorium formation rate **(C)**. Conidial germination dynamics were observed and germination rates were recorded after 48 h of continuous incubation at 28°C in the dark under moisturizing conditions, with attached cells being produced during spore germination. Adherent cell formation was observed continuously for 24 h at 28°C in the dark and the rate of formation was recorded, bar = 10 μm.
