# Supplementary figures and images for: Monitoring peroxisome dynamics using enhanced green fluorescent protein labeling in Alternaria alternata
Source: Front Microbiol. 2022 Oct 25;13:1017352. doi: 10.3389/fmicb.2022.1017352 (PMC9640759; doi:10.3389/fmicb.2022.1017352)

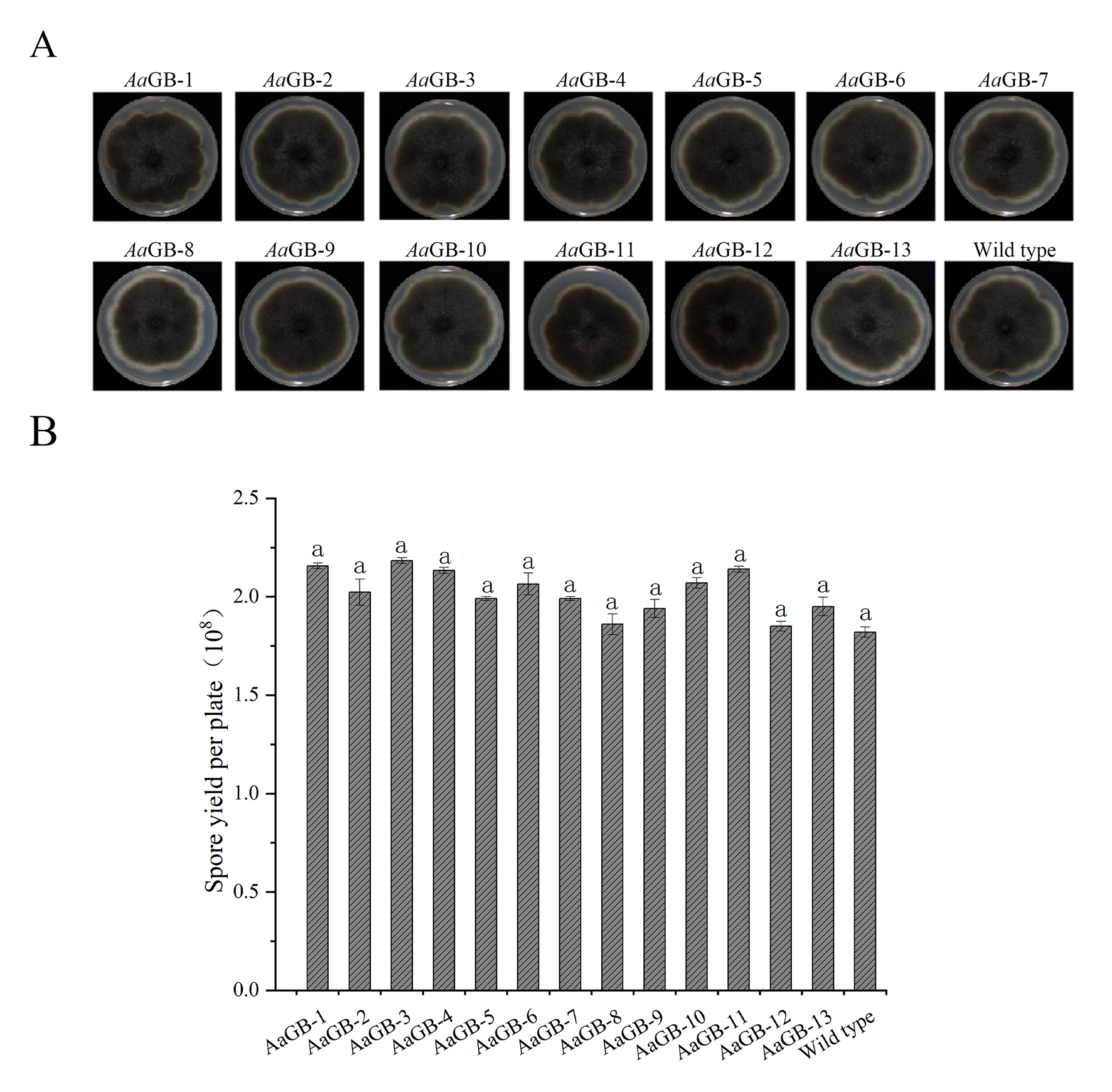

Supplement: Supplementary Figure 1 — PHMGB-Aa transformant colonies and wild-type morphology (A) and their sporulation (B). These colonies and wild-type were obtained by culturing in CM medium under dark conditions of 25°C for 7 days. [file Image_1.TIF]

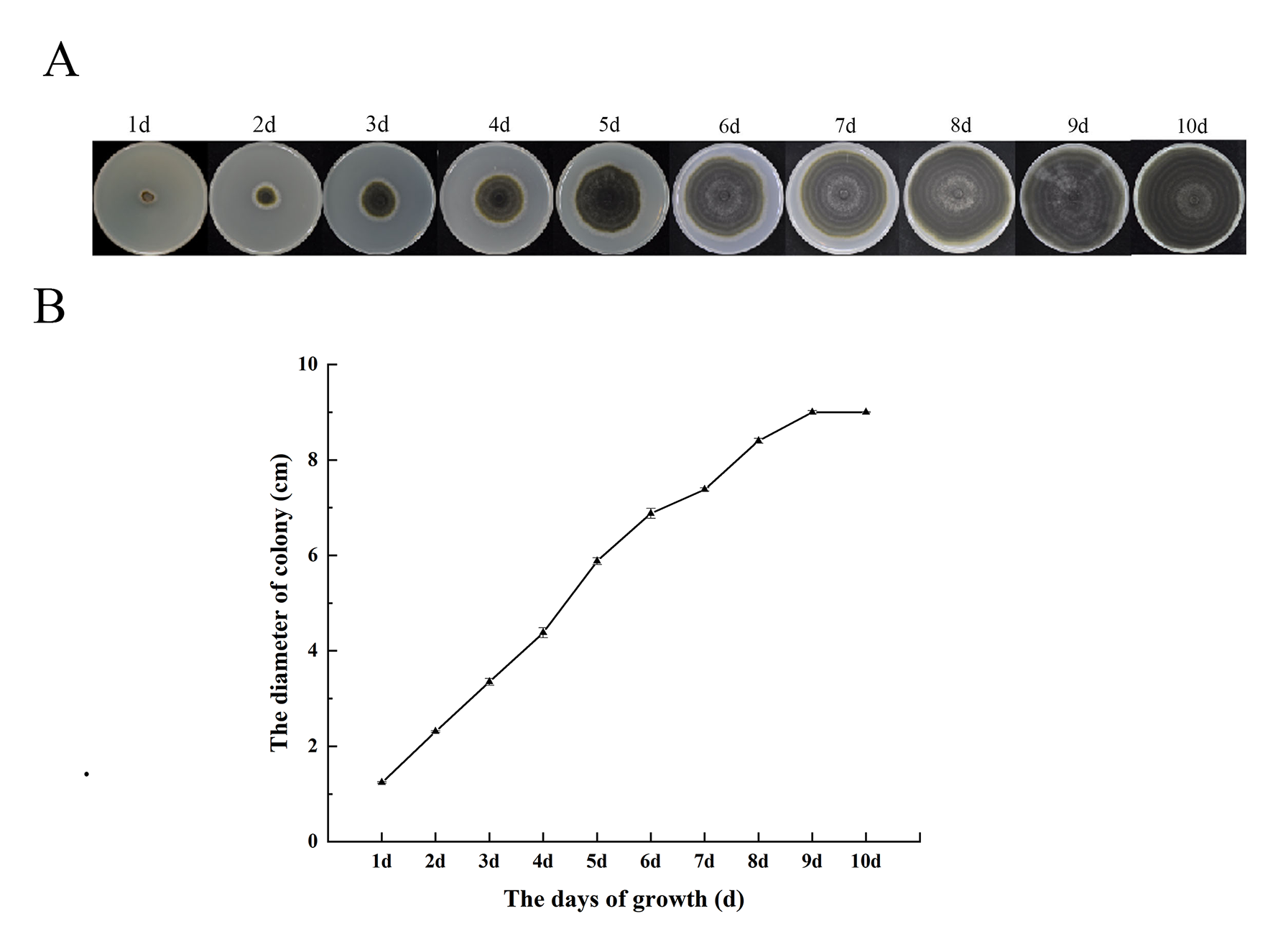

Supplement: Supplementary Figure 2 — AaGB colony growth morphology changes (A) and growth diameter of the colony (B). Observed and recorded continuously for 10 days at 28°C under light conditions. [file Image_2.TIF]

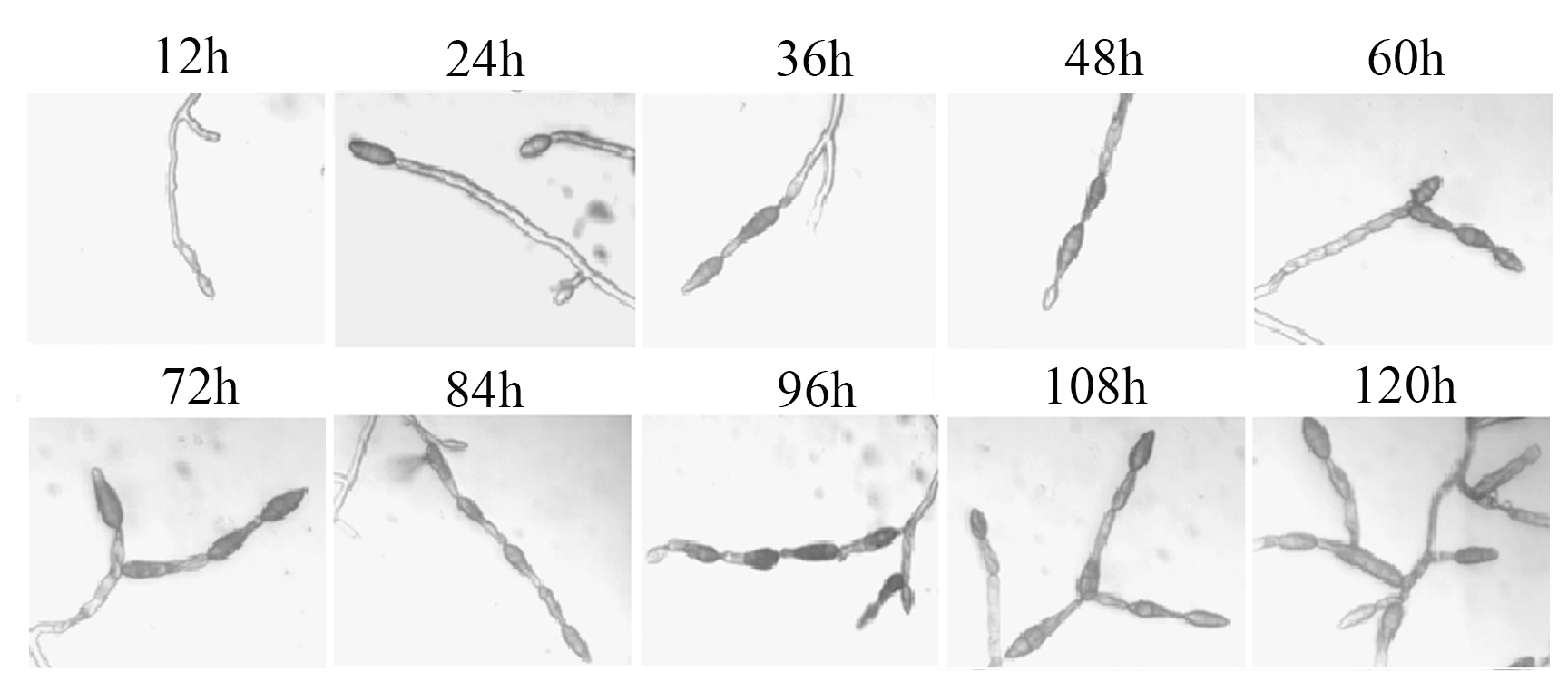

Supplement: Supplementary Figure 3 — AaGB transformant spore growth dynamic. The growth dynamics of spores in 5 days under 25°C light conditions were observed and recorded. [file Image_3.TIF]

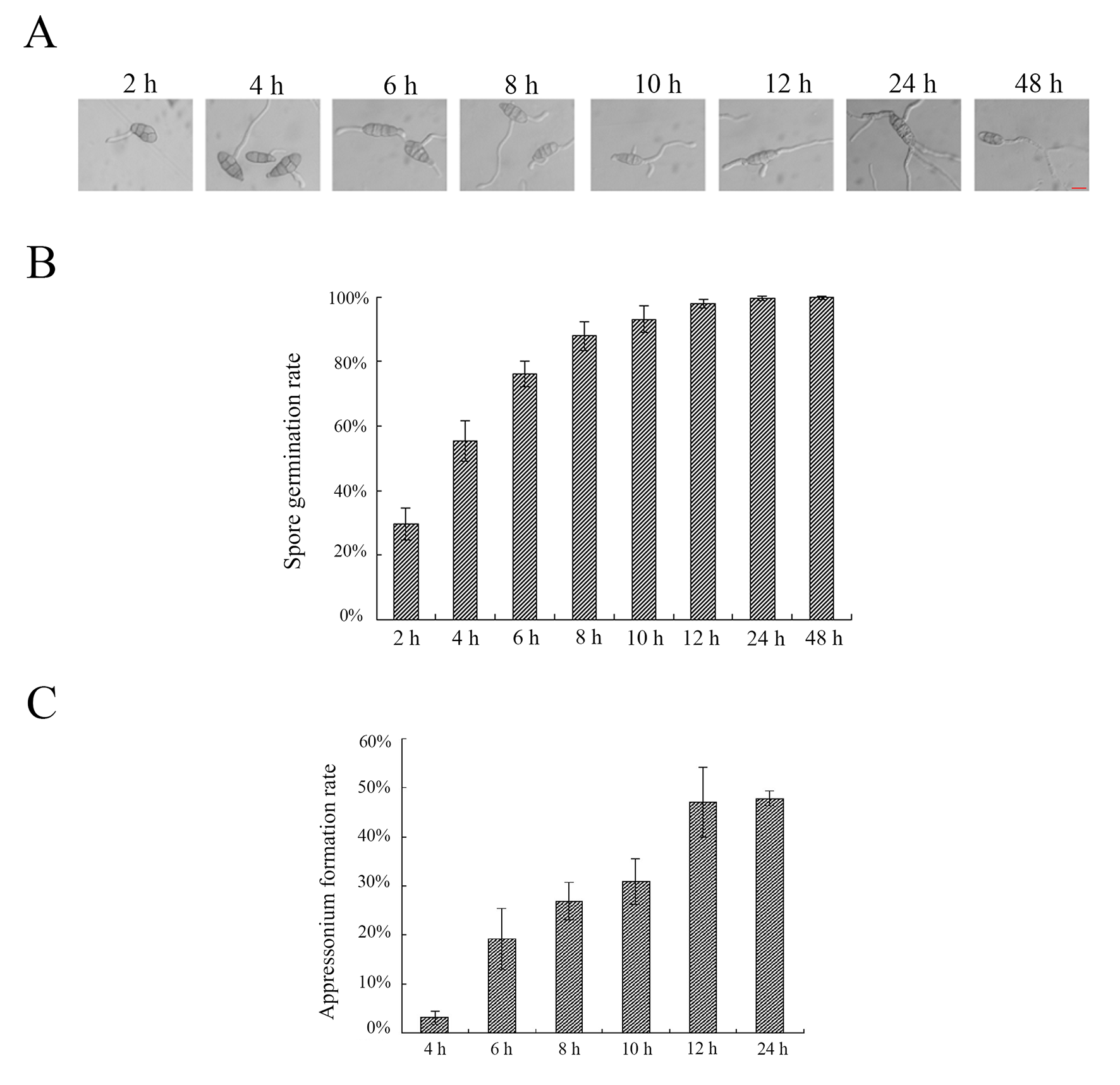

Supplement: Supplementary Figure 4 — Observation of the dynamics of AaGB conidial germination (A) and its germination rate (B), appressorium formation rate (C). Conidial germination dynamics were observed and germination rates were recorded after 48 h of continuous incubation at 28°C in the dark under moisturizing conditions, with attached cells being produced during spore germination. Adherent cell formation was observed continuously for 24 h at 28°C in the dark and the rate of formation was recorded, bar = 10 μm. [file Image_4.TIF]
